# 2485. *Candida auris* Hospital Cluster: Were Our Unwelcome Guests Driven Away by the COVID-19 Delta Surge?

**DOI:** 10.1093/ofid/ofad500.2103

**Published:** 2023-11-27

**Authors:** Jyoti Somani, Nazira Muhammad Fauzi, Revathi Sridhar, Hwang Ching Chan, Isaac Taoyang Low, Ka Lip Chew, Dale Fisher

**Affiliations:** National University Hospital, Singapore, Singapore; National University Hospital Singapore, Singapore, Not Applicable, Singapore; National University Hospital Singapore, Singapore, Not Applicable, Singapore; National University Hospital, Singapore, Singapore; National University Hospital Singapore, Singapore, Not Applicable, Singapore; National University Hospital, Singapore, Singapore, Not Applicable, Singapore; National University Hospital Singapore, Singapore, Not Applicable, Singapore

## Abstract

**Background:**

*Candida auris* (CA) is an MDR pathogen that persists in the environment, causing outbreaks. NUH is an academic medical centre in Singapore. We describe a cluster of CA cases from April 2019 – October 2021. Our last case was during our Delta surge, just before we changed our routine inpatient cleaning to bleach based from quarternary-ammonium.

**Figure:**
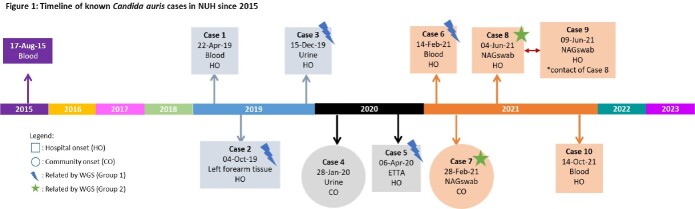

**Methods:**

Any CA from the micro lab is escalated to the Infection Prevention Team to initiate isolation, cleaning, and contact tracing (CT) up to 30 days prior to positive cultures. In September 2020, we started enhanced surveillance via NAG (Nasal, axilla and groin) swabs in all new CRE (Carbapenem-Resistant Enterobacterales) patients. Surveillance cultures used sabouraud dulcitol agar with 10% saline incubated at 40°C. Identification and susceptibility testing done using Bruker MALDI Biotyper and Etest (Biomerieux). Hospital onset (HO) is defined as positive cultures sent after day 3 of admission. CA isolates were sent for whole genome sequencing (WGS).

**Results:**

The 10 cases included 7 from clinical isolates, 2 from enhanced surveillance and 1 from CT. 8 were HO. 7 deaths were during the CA admission, but only 1 due to CA, and 1 occurred > 60 days after positive culture [Table 1]. Of 231 contacts swabbed all were negative, except for Case 8, in whom one contact (Case 9) was positive. Positive yield from enhanced surveillance of CRE patients, was 0.84% (2/239). WGS results indicated all except Case 9 belonged to Clade I with Y132F mutation in ERG111 gene [Table 2]. Cases 2, 3, 5, 6 were closely related with 2-7 SNPs difference, but spaced in years. Cases 7 and 8 differed by 3 SNPs hence, closely related. There were 2 related clones being transmitted, and 4 unrelated sub-types of CA. Case 8 and 9 were not related, though 9 was a contact of 8. Possible links were shared equipment or admission to a common room however, admissions were either non-overlapping or non-successive [Table 3]. Case 5 was admitted to the same room after a long stay by Case 3, with one patient in between. No links found between Cases 7 and 8.

**Figure:**
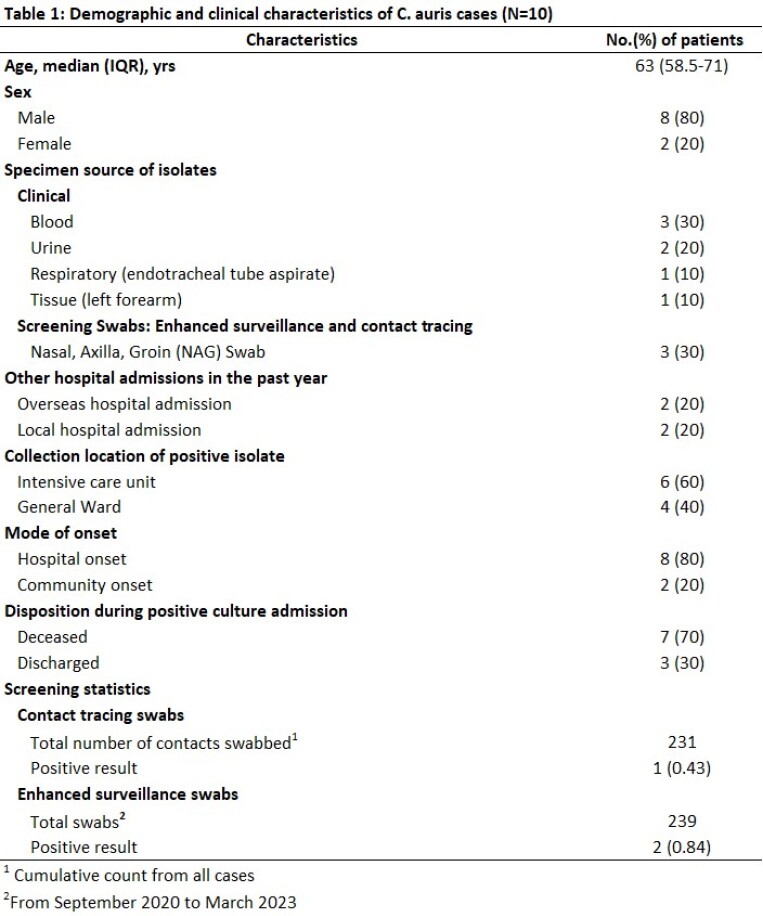

**Figure:**
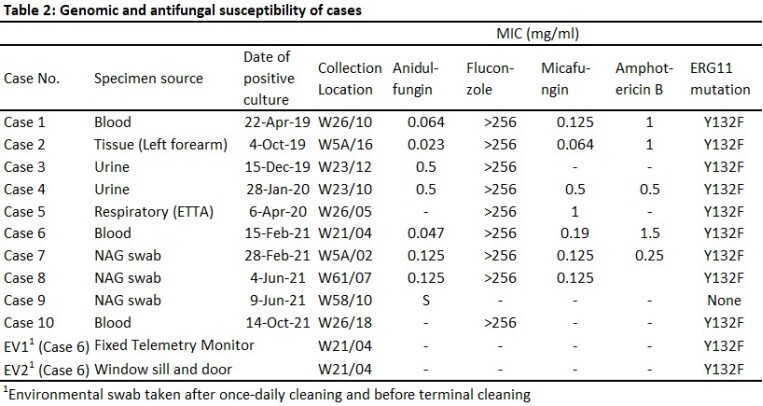

**Figure:**
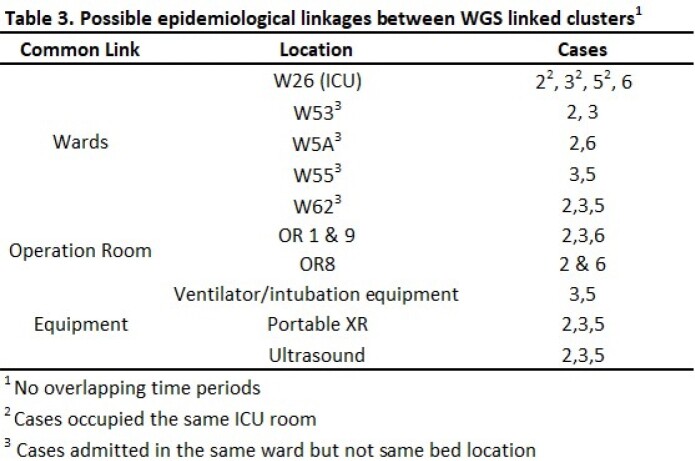

**Conclusion:**

WGS results suggest that various clones of CA remain in the environment as reservoirs, and related cases are not often spatiotemporally related. There have been no CA cases since Oct 2021, possibly due to the change to bleach-based cleaner during our Delta surge.

**Disclosures:**

**All Authors**: No reported disclosures

